# Remarks on 177 Operations for Entropium and Trichiasis

**Published:** 1883-06

**Authors:** F. C. Hotz

**Affiliations:** Chicago, Ill.


### Selections.

---

REMARKS ON 177 OPERATIONS FOR ENTROPIUM AND TRICHIASIS. BY F. C. HOTZ, M. D., Chicago, Ill.

The author of a new operation or of a modification of an old method ought, after a few years, to give a candid report of his experience. If his operation has proven a success, the profession will readily adopt it; if a failure, it is no less important for the profession to know it. But sometimes we do not learn of the results because they have not been very brilliant. For fear, therefore, my silence might be misinterpreted, I wish to give a brief report of the results I attained in the past four years by the operation for entropium and trichiasis published in volume viii, No. 2 of these Archives. And at the same time I wish to supplement my former paper by a few remarks on the operation.

The essential features of my operations are these: The skin of the eyelid is incised transversely in the line of the upper border of the tarsus of the upper lid (or along the lower border of the tarsus of the lower lid); the muscular layer covering that border of the tarsus is excised (about 3 to 4 *mm.* in width); and the cutaneous edges of the incision are brought in close adaptation with the cartilage by sutures which are passed right through the border of the tarsus and the tarso-orbital fascia.

The success of the operation depends a great deal on the correct incision; it is, therefore, of paramount importance that the surgeon has a clear idea of this point. Now, I have several times seen my incision described as being made 2 *mm.* above the free border of the eyelid. Nothing could be more erroneous than this statement. Perhaps the error was caused by my saying (*l. c.*, p. 255): "I apply the point of a scalpel at a point 2 *mm.*

above the inner canthus and draw a horizontal incision across the lid to a point 2 *mm.* above the external canthus." He, of course, who had read the preceding passages with attention, and only looked at the wood-cut accompanying the description, could never get so false a conception ; for it was expressly remarked the incision should follow, as closely as possible, that furrow which is the border line between the skin of the eyelid and the supratarsal integument. This furrow (in the upper lid) describes a curve beginning 2 *mm.* above the inner canthus, and ending 2 *mm.* above the outer canthus, but its center is from 6 to 8, and sometimes even 10 *mm.* removed from the cilia. But as the free mobility of the tarsal skin makes it exceedingly difficult to make the curved incision with the desired precision and nicety, I suggested temporarily to change the curve into a straight line: "I seize, between thumb and forefinger, or with a pair of forceps,* the center of the free edge of the lid and draw it downward until the skin is moderately stretched. As both the commissures are fastened to the bones and thus rendered immobile, the center of the lid alone is affected by this traction, the free edge obtains a convexity downward, while the curved furrow which markes the upper border of the cartilage is reduced to a straight horizontal line." In other words, the center of the furrow and the center of the ciliary edge are displaced downward at the same rate, their original distance being uninfluenced by the traction. Consequently, an incision which follows that furrow while temporarily drawn out to a horizontal line, begins and ends 2 *mm.* above the commissures, but its center recedes from the lid border 6, 8 or 10 *mm*, according to the varying width of the tarsus. This incision sacrifices nothing of the tarsal skin; the lid retains after the operation its original integument, and in consequence thereof its natural appearance and unimpeded movements, so that this operation, judged by its cosmetic effect, may be considered superior to all other procedures.

Some surgeons, I have noticed, make the incision too near the cilia, because they seem still to believe the eversion of the inverted lid requires a strong traction, obtained from the forcibly

*If the eyelashes are too short to offer a good hold to the fingers.

stretched skin. I explained on former occasions why this opinion is wrong and should be dropped, and my numerous operations are the best practical evidence in support of my theory, that a very small force and a slight tension of the skin are sufficient for the cure of entropium, provided the tension proceed from a fixed point, such as the upper border of the tarsus offers.

When the skin of the eyelid is very flaccid and wrinkled, or when the excision of a wedge-shaped piece of cartilage is contemplated, whereby the width of the cartilage would be diminished, only in these cases may the incision be made below the oft-mentioned furrow to avoid having a super-abundance of skin after the operation. But should the incision be made in the furrow and the tarsal skin afterward be found super-abundant, the mistake can easily be rectified by the removal of a narrow strip of skin from the lower border of the wound. At all events, it is better to make a mistake in this rather than in the opposite direction; for if the tarsal skin turns out to be too wide, it can easily be shortened; but if too short, it cannot be lengthened. An evil consequence necessarily resulting from the short-cut skin is the great difficulty of uniting the edge of the tarsal skin with the upper border of the tarsus. Either it cannot be done at all, or only by unduly stretching the skin. In the first instance, the sutures cannot pass through the border of the tarsus at all; and in the second case, the tension being too great, they are likely to cut through prematurely, allowing the abnormally stretched piece of skin to recover its natural state, in which it is wholly insufficient to cover the whole surface of the lid and to unite with the upper border of the cartilage.

*Only when the tarsal skin becomes firmly united with the border of the cartilage is the permanent success of the operation insured.* In the highest degrees of entropium and trichiasis, with much contraction and incurvation of the tarsus, it is a common occurrence that the upper border is as much curved in as the lower; and the edge we see after removing the muscular layer is not the real upper line of the cartilage, but the angle of incurvation. In order to avoid mistaking the apparent for the real upper border, and to make sure that the sutures hold the real border, I have adopted the following plan: After the needle has been drawn through the cut edge of the tarsal skin, I prick it into the cartilage near its upper border, and with it lift the lid off from the eyeball far enough to push one prong of a finely-toothed forceps from above downward between the lid and eyeball, while the other prong passes down in front of the cartilage. In this way the upper border of the tarsal cartilage must get between the prongs of the forceps, which hold it so well that the needle can transfix it with the greatest precision and safety at the properly selected point. I have never seen any untoward symptoms arise from this manipulation.

Canthotomy and canthoplasty were often performed in connection with my operation, and in all cases presenting a thickened and contracted tarsus, Streatfield's grooving was combined with the operation. The splitting of the tarsal edge has been discarded as useless; its immediate effect upon the position of the eyelashes is very nice, to be sure, and lasts about as long as the gap in the lid border. When the gap is filled with granulations, the inevitable contraction of the cicatrical tissues will do its work so thoroughly that the gap is contracted to a fine linear scar in the tarsal edge, and the eyelashes, yielding to this traction of the scar, begin to bend down again. The same objection applies to Ammon's and similar incisions made behind and parallel with the tarsal edge into the conjunctival surface of the lid.

And now a few words about the results of my operations. In the space of four years (Sept., 1878, to Oct., 1882,) I have performed 177 operations for entropium and trichiasis, of which 142 were on the upper lid and 35 upon the lower lid. Among the cases there were all sorts and grades of inversion represented. Although I have kept a careful record of all the cases, noting the state of the lids before the operation, the healing process, the result at the time when the patients were discharged, as well as the condition of the lids at subsequent examinations (when such opportunity offered itself), I shall not annoy the readers by statistical tables, but content myself with saying that the results generally have been satisfactory in the highest degree, and that I value the operation the more, the oftener I have performed it.

Among all the 177 cases there was not one in which the operation failed directly. In several cases, I admit, the eyelashes were not everted as much as I expected after the sutures were tied; but upon re-opening the sutures—as I always did under these circumstances—I found the imperfect effect to be due to faulty application of one suture or the other. Either the thread had not passed through the border of the cartilage at all, or only so superficially that it cut through when the knot was tightened. The readjustment of the faulty sutures always had the desired effect upon the position of the cilia. "Haste makes waste" and is not compatible with my operation which will therefore not likely be a great success in the hands of those who can spare but five or ten minutes for a lid operation.

My tables record 12 relapses in 142 operations for entropium of the upper lids. In 3 of the 12 cases partial entropium had occurred near the canthus; these cases belong to the first stage of the operation, when I yet thought the cilia nearest to the canthus could be everted permanently, without the deep sutures, by the removal of small triangles of integument. Since I have dropped this procedure and employed the deep sutures alone, I have not seen a relapse of this kind.

Four other instances of relapse also belong to the initial period of this operation. The lids presented the highest degree of inversion, and very marked incurvation of the cartilage, for which at present I consider grooving an absolute necessity. For the sake of an experiment, however, I did not groove the cartilages of those lids, because I was curious to see how much my method alone, without the aid of other means, could achieve in these worst forms of entropium; and I was gratified to see that even under such unfavorable circumstances, the eyelashes could be turned enough to clear the eyeball without one particle of skin having been excised; but the eversion of the tarsal edge was not complete so as to prevent a relapse. In repeating the operation the tarsi were grooved, and the results obtained which two years later were still perfect.

The eighth case of relapse occurred in a patient suffering from inveterate lues (which fact was not known to us at the time of the operation); the wound healed very imperfectly, and re-opened after two weeks to reveal extensive ulceration, proceeding from the groove in the tarsus. Relapses 9 and 10 occurred in a young lady who returned to her home in Minnesota ten days after the operation. When she left, the lids were in a very nice condition, but at home (possibly from the severe cold weather in January), the lids became swollen, painful, and extremely sensitive to cold air. This state lasted about four weeks, and when the patient came back to the city her eyelids were still very tender upon pressure and sensitive to cold. It seemed to me that an inflammation and softening of the tarsus had taken place, and that under these circumstances the tarsal edge had again yielded to the traction of the very atrophic conjunctiva. In two instances, finally, the retro-tarsal fold of the conjunctiva was obliterated, and the height of the cartilage reduced to five *mm.* by atrophy of the conjunctiva and cartilage. In such cases the prospect for permanent improvement is very dubious ; the fornix being obliterated, the lid is tied down, as it were, to the eyeball, which drags it along in its rotation. This continuous dragging creates chronic irritation and inflammation of the tissues of the eyelid, and in this way leads to further shrinkage of the component parts of the lid.

This extreme shrinkage of conjunctiva and tarsus is a very important factor in the results of the operation upon the lower lid, and has rendered a permanent relief impossible in three cases. In two other cases, however, the relapse must be charged to a faulty application of the sutures. In repeating the operation, it was discovered that the tarsus was unusually broad, and its lower portion was strongly bent inward, so much so, that the angle of the bend had been taken for the lower border of the cartilage, and the sutures been put in the wrong place.

As I could not examine every case at some later period, I can of course, not claim that these seventeen cases represent the actual percentage of relapses ; on the contrary, I have no doubt that their number is greater, because I have operated on other cases under the same conditions as those which were followed by relapses. I mentioned the above cases simply to point out some of the influences which can affect the result of the operation, and to show that some of the causes have no particular relation to the operation I employed, but will have the same bad effect upon the results of any other method. From my whole experience, I dare say that relapses will occur comparatively seldom, provided the operation be done with care and accuracy.

Among my cases there were fourteen upper eyelids upon which other operative methods had been tried for the relief of entropium. In the most cases Arlt's operation had been performed; and it was interesting to observe how seriously the movements of the upper eyelid are impeded by the removal of the tarsal skin, and that the degree of the disturbance stood in close relation to the size of the excised integument. In these cases we must take particular pains to make the incision in a line with the upper border of the tarsus, because after the transverse incision is made, the skin which was strctched and dragged out of place by the previous operation will shrink so mueh that there will not be enough to cover the whole lid with if the incision be made lower than the upper border of the tarsus. The skin must be dissected off from the tarsus clear down to the eyelashes by dividing all the cicatricial bands connecting the two parts; the cartilage being grooved, the sutures are put in. In this way I succeeded in relieving the entropium as well as in rcstoring to the eyelid its free and easy movements.

Operations for entropium producing perpendicular scars upon the eyelid are a gross offense against good taste and cosmetic laws, and the sooner they are excluded from the sphere of legitimate surgery the better. Twice I had occasion, in operating for entropium, to relieve the lid of the hideous deformity produced by such perpendicular scars.

With a few exceptions, the after-treatment consisted in the application of wet compresses during the first twenty-four hours; in six cases, borated cotton was used instead. First union was obtained ın 140 cases; in thirty-seven cases pus showed itself in the sutures on the second or third day. The constitutional condition of the patients seemed to influence the healing process more than any other cause; among forty-three patients whose constitution was weakened by scrofula, anæmia, malnutrition, etc., suppuration occurred twenty-four times, while among the

134 robust patients the healing was disturbed in seven cases only.

Reviewing the results of my operations after a four-years' trial, I can say that they have well sustained and corroborated my former statement, that this operation possesses the following advantages and meritorious features:

1. That it accomplishes its purpose (relief of entropium) without the slightest destruction of skin.

2. That for this reason it can be employed in cases where other methods are impracticable on account of excessive shortness of the integument of the lid.

3. That it does not mutilate the lid or in any way interfere with its movements.

4. That in a case of relapse it can be repeated without in the least disturbing the natural appearance of the lid.

5. That the tension by which the inverted eyelashes are turned back to their normal position is rendered independent of the movements of the lid, because the distance between the two points upon which the tension is to exert its influence, viz., the upper border of the tarsus and the free edge of the lid, remains the same whether the lid is raised or dropped; while where the entropium is relieved by the shortening of the integument, the tension is subjected to considerable variations, because it is regulated by the distance of the free edge of the lid from the supra-orbital margin. This distance varies with the movements of the eyelid; it is greatest when the lid is closed, and therefore in this position of the lid the tension exerts its greatest influence upon the tarsal edge; but when the lid is raised, its free edge approaches the supra-orbital margin; consequently, the integument between these two points becomes relaxed, and the tension is greatly diminished, and may even be reduced to zero. Under these circumstances, therefore, the upper lid can appear everted when closed, and inverted when open.—*Archives of Ophthalmology*, *December*, 1882.

